# Temperature-Dependent Photoluminescent Properties of PbSe Nanoplatelets

**DOI:** 10.3390/nano10122570

**Published:** 2020-12-21

**Authors:** Ivan Skurlov, Anastasiia Sokolova, Tom Galle, Sergei Cherevkov, Elena Ushakova, Alexander Baranov, Vladimir Lesnyak, Anatoly Fedorov, Aleksandr Litvin

**Affiliations:** 1 Center of Information Optical Technology, The Laboratory “Optics of Quantum Nanostructures”, ITMO University, 49 Kronverksky Pr., St. Petersburg 197101, Russia; sky_id@itmo.ru (I.S.); avsokolova@itmo.ru (A.S.); s.cherevkov@itmo.ru (S.C.); elena.ushakova@itmo.ru (E.U.); a_v_baranov@itmo.ru (A.B.); a_v_fedorov@itmo.ru (A.F.); 2 Physical Chemistry, TU Dresden, Zellescher Weg 19, 01069 Dresden, Germany; tgalle@cech05.chm.tu-dresden.de (T.G.); vladimir.lesnyak1@tu-dresden.de (V.L.)

**Keywords:** nanoplatelets, 2D nanomaterials, lead selenide, cation exchange, photoluminescence, temperature dependent photoluminescence

## Abstract

Semiconductor colloidal nanoplatelets (NPLs) are a promising new class of nanostructures that can bring much impact on lightning technologies, light-emitting diodes (LED), and laser fabrication. Indeed, great progress has been made in optimizing the optical properties of the NPLs for the visible spectral range, which has already made the implementation of a number of effective devices on their basis possible. To date, state-of-the-art near-infrared (NIR)-emitting NPLs are significantly inferior to their visible-range counterparts, although it would be fair to say that they received significantly less research attention so far. In this study, we report a comprehensive analysis of steady-state and time-dependent photoluminescence (PL) properties of four monolayered (ML) PbSe NPLs. The PL measurements are performed in a temperature range of 78–300 K, and their results are compared to those obtained for CdSe NPLs and PbSe quantum dots (QDs). We show that multiple emissive states, both band-edge and trap-related, are responsible for the formation of the NPLs’ PL band. We demonstrate that the widening of the PL band is caused by the inhomogeneous broadening rather than homogeneous one, and analyze the possible contributions to PL broadening.

## 1. Introduction

Colloidal nanoplatelets (NPLs) are a new type of semiconductor nanomaterials with superior light-emitting properties which are highly demanded for displays and light-emitting diodes (LED) [1,2,3,4]. Due to their thickness-dependent optical properties and the possibility of synthesis of NPLs of strictly specified thickness, extremely narrow photoluminescence (PL) bandwidths are observed. Further improvement of classical core NPLs can achieved by heterostructuring [5]. Recently, a record external quantum efficiency of 19.2% was achieved for the red-emitting LED with CdSe/Cd_0.25_Zn_0.75_S active layer [6]. Importantly, a turn-on voltage as low as 1.63 V has recently been demonstrated for NPLs-based LEDs [7]. Furthermore, Wu and co-workers have shown the possibility of room-temperature lasing with core-shell NPLs [8].

Near-infrared (NIR) emitting two-dimensional colloidal semiconductor nanomaterials were first synthesized by Weller’s group using the oriented attachment method [9]. PbS sheets with a thickness of 2.2 nm had lateral dimensions of hundreds of nm and demonstrated a wide PL band centered at 790 nm. Since that time, PbS [10,11,12,13,14,15], PbSe [16,17], PbSe_1−*x*_S*_x_* [18], and HgTe [19,20] NIR-emitting NPLs were synthesized using both oriented attachment and cation exchange procedures. A different strategy to obtain NIR-emitting NPLs was to dope visible-emitting CdSe NPLs with Ag [21,22] or Cu [23]. Curiously, that HgTe NIR-emitting NPLs exhibit optical properties typical for this class of objects, including narrow PL width and short PL lifetimes. An important limitation is the fact that no HgTe NPLs with a PL signal beyond 0.9 µm were reported. At the same time, lead chalcogenide-based NPLs normally possess wide PL bands, while PL decay times span a broad range of values from tens of ns to µs. The only report for narrow NIR emission from Pb-containing NPLs has been made by Khan and co-workers who used a single precursor (lead octadecylxanthate) for oriented attachment synthesis [11].

In order to understand the prospects of using semiconductor colloidal NPLs in the NIR to build LEDs with broadly tunable emission wavelengths, it is necessary to understand the mechanisms leading to the broadening of spectral lines in Pb chalcogenide-based NPLs. The study of temperature dependencies of PL parameters is a versatile tool to understand the energy structure of nanomaterials and mechanisms of radiative and non-radiative recombination [24,25,26,27,28]. The studies of PL temperature dependencies have recently been reported for CdSe NPLs [29,30,31,32,33,34,35,36] and their core@shell and core/crown@shell heterostructures [37]. Ithurria et al. demonstrated shortening of PL lifetimes for 7-multilayer (ML)-thick CdSe NPLs with temperature decrease [29]. This observation was in stark contrast to that observed for the 0D counterparts and was ascribed to the effect of a giant oscillator strength transition, which was previously observed in semiconductor quantum wells [38]. This observation was further supported by Tessier et al. who studied the temperature dependencies of PL from single CdSe NPLs and reported an ultrafast recombination time of 200 ps at 20 K [30]. At extra-low temperatures, the splitting into dark and bright excitonic levels was studied both experimentally and theoretically [31,35]. It was further demonstrated that the organization of anisotropic NPLs in oriented stacks influences the observed temperature dependencies of PL parameters [32,33]. Although great efforts were made to study the PL behavior of CdSe NPLs in different temperature regimes including elevated [34,36], there is a lack of such investigations for other types of semiconductor NPLs. Carrying out such studies for lead chalcogenide-based NIR-emitting NPLs is especially important for understanding the mechanisms of radiative recombination and assessing the nature of the broadening of their PL bands.

Investigation of the temperature dependences of the PL is not only of fundamental interest for understanding the mechanisms of energy relaxation, but also makes it possible to analyze the possibility of practical application of nanomaterials in such applications as luminescence nanothermometry and thermal sensing [39,40] NIR-emitting nanoparticles are of special interest due to the possibility of studying live tissues [41]. A high quantum yield of NIR PL from 0D and 2D lead chalcogenide-based nanostructures combined with large and variable coefficients of a temperature shift of PL peak position make them a promising candidate to compete with well-known nanothermometers based on lanthanide-doped nanocrystals [42,43].

## 2. Materials and Methods

### 2.1. Synthesis of CdSe and PbSe NPLs

CdSe NPLs synthesis: A 50 mL three-neck flask was loaded with CdO (70 mg, 0.545 mmol, Sigma-Aldrich, St. Louis, MO, USA) and myristic acid (340 mg, 1.49 mmol, Sigma-Aldrich, St. Louis, MO, USA). 27 mL of 1-octadecene (ODE, Sigma-Aldrich, St. Louis, MO, USA) was added and the mixture was degassed for 30 min at 100 °C. Then, the flask was filled with nitrogen and the temperature was increased to 285 °C where the mixture was kept until the complete dissolution of the CdO was apparent, resulting in a clear solution. The flask was cooled down to 100 °C and degassed again, in order to remove water from the reaction mixture. In the meantime, a Se-precursor was prepared in a nitrogen-filled glovebox by mixing Se powder (24 mg, 0.31 mmol, Chempur, Karlsruhe, Germany) with 3 mL of ODE in a glass vial followed by an ultra-sonication treatment for 30 min. This dispersion was then added into the nitrogen-filled three-neck flask at 100 °C. Hereafter, the mixture was heated to 240 °C. The NPL-growth was triggered by the swift addition of Cd(OAc)_2_·2H_2_O (160 mg, 0.6 mmol, Merck, Darmstadt, Germany) at 195 °C. When the growth temperature of 240 °C was reached, a solution of SeO_2_ (40 mg, 0.36 mmol) in 5 mL of ODE, which was prepared by stirring at 200 °C until a clear orange solution formed, was injected at a rate of 25 mL/h for 10 min. When the injection finished, the flask was cooled down using a water bath and 2 mL of oleic acid were added at approximately 160 °C. The obtained four monolayered (ML) NPLs were precipitated using a hexane/ethanol mixture (3:1—vol.) and subsequent centrifugation. The precipitated NPLs were dispersed in 5 mL of toluene. The transmission electron microscopy (TEM) image of CdSe NPLs are shown in Figure S1A (Supplementary Materials). The top- and bottom facets are capped with Cd-atoms, resulting in 5 layers of Cd-atoms and four layers of Se-atoms along the thickness of the NPLs. There is no size dispersity along the z-direction and the thickness is 1.2 nm. The lateral dimensions, however, show significant size dispersity of roughly 15%. Manual measurement of 28 NPLs using ‘ImageJ’ results in an average size of 28 × 15 nm.

### 2.2. Cation Exchange of CdSe to PbSe

To obtain PbSe NPLs, we used a particular case of the synthetic route that we have recently described in [17]. Briefly, 50% of a CdSe NPL sample prepared as described above was used for the cation exchange. The CdSe NPLs were precipitated with ethanol and re-dispersed in toluene, then loaded into a syringe. In a 25 mL three-neck-flask PbBr_2_ (125 mg, 0.34 mmol) was mixed with 14 mL of ODE and 2 mL of oleylamine and degassed at 100 °C for 20 min. The temperature was lowered to 80 °C and the flask filled with nitrogen, before the CdSe NPLs dispersion was injected rapidly. The cation exchange was allowed to proceed for 5 h before the flask was brought to room-temperature using a water bath. The complete reaction mixture was transferred into a centrifuge tube containing toluene (3 mL), ethanol (12 mL), and oleic acid (1 mL). During the cation exchange reaction, the NPLs undergo some etching along their edges, clearly visible in bright-field TEM images shown in Figure S1B,C (Supplementary Materials). The determination of their lateral sizes via direct measurement is therefore not feasible, but the aforementioned size can be treated as the upper bound. We have no reason to believe that their thickness is changed systematically during the reaction, however, it is plausible that additional defects along the z-direction can be created during the cation exchange. The NPLs were precipitated by centrifugation, the supernatant was discarded, and the precipitate dispersed in tetrachloroethylene. In order to perform this cleaning procedure under inert-atmosphere nitrogen-flushed glass vials with septum and syringes were used to transfer the reaction mixture into the nitrogen-filled glovebox. The NPLs were stored at room-temperature in the dark.

### 2.3. Characterization

Transmission electron microscopy (TEM) imaging was carried out on a JEOL JEM-1400 microscope (JEOL Ltd., Tokyo, Japan) equipped with a thermionic gun (W filament) working at 120 kV accelerating voltage. The samples were prepared by diluting the NPL dispersions and drop-casting onto a carbon-coated copper grid with subsequent evaporation of the solvent. 

All spectroscopic studies were carried out in ambient atmosphere. For absorption and PL measurements NPLs were dissolved in tetrachloroethylene, which is transparent in the NIR. Absorption was measured using Shimadzu UV3600 spectrophotometer (Shimadzu, Kyoto, Japan). NIR PL spectra were measured using custom-built setup [44] with an excitation at 633 nm. PL spectra of CdSe NPLs in the visible range were measured using 405 nm continuous wave laser (Lasever Inc., Ningbo, China) as an excitation source and Si-based CCD-camera (Andor iDus 401, Andor, Belfast, Northern Ireland) as a detector. All spectral data were corrected by the spectral sensitivity of the setup obtained by using the standard spectrum of an ideal black body [45]. Time-resolved photoluminescence (TRPL) measurements in the NIR were carried out using a single-photon avalanche diode (Micro Photon Devices, Bolzano, Italy) synchronized with 635 nm pulsed (~100 ps, 25 kHz) laser as an excitation source [46]. To study the temperature dependencies of PL parameters, the NPLs were embedded both into poly(methyl methacrylate) matrix and in a special filter paper [47,48]. For each medium and each sample, the temperature dependencies were obtained at least twice. Both matrices gave similar qualitative dependencies, and the data obtained by using a filter paper are presented in the manuscript. A low concentration solution of NPLs was dropped on the piece of porous paper (Sartorius 388 grade, pore diameter > 20 µm, Sartorius, Göttingen, Germany). After drying, the samples were transferred into the nitrogen cooled cryostat (Linkam THMS 600) for further measurement. Raman scattering was measured using micro-RS InVia spectrometer (Renishaw, Wotton-under-Edge, UK) with Renishaw Streamline™ technology. A HeNe laser (wavelength 633 nm, PLASMA, Ryazan, Russia) was used as an excitation source. Excitation signal was cut off by the edgepass filter. Samples were prepared via drop-casting the stock solution onto the cleaned glass substrate and the solvent was allowed to evaporate.

## 3. Results and Discussion

**Figure 1:**
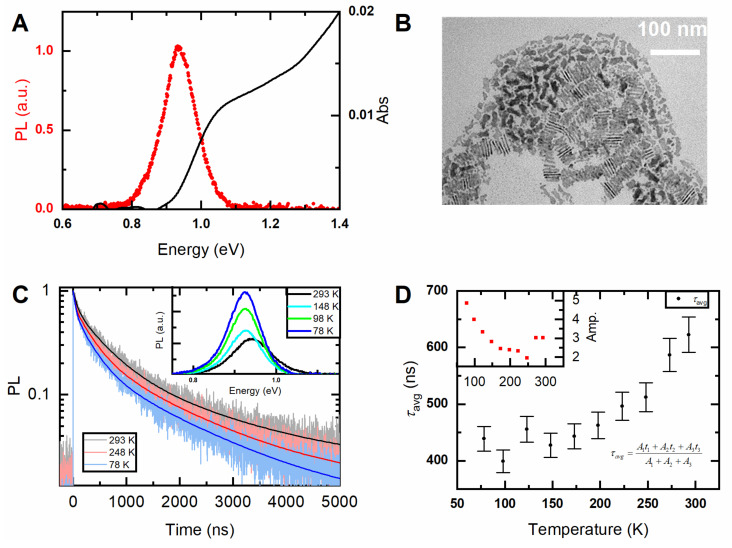
(**A**) Absorption (black line) and photoluminescence (PL) (red dots) spectra of four monolayered (ML) PbSe nanoplatelets (NPLs) in tetrachloroethylene; (**B**) transmission electron microscopy (TEM) image of 4 ML PbSe NPLs (**C**) PL decay curves recorded at different temperatures, inset—PL spectra obtained in 78–300 K temperature range (**D**) averaged PL decay times vs. temperature, inset—integrated PL intensity vs. temperature.

Typical PL and absorption spectra of 4 ML PbSe NPLs are shown in Figure 1A, corresponding TEM image is demonstrated in Figure 1B. The intense NIR PL band is centered at 0.93 eV (1330 nm) with full width at half maximum (FWHM) of 116 meV. Steady-state and kinetic PL measurements were performed in a temperature range of 78–300 K. PL decay curves and spectra, recorded at different temperatures are shown in Figure 1C. PL decay curves of NPLs were fitted by 3-exponential decay function. The average lifetimes at each temperature was calculated using amplitude averaging:

(1) $$I(t)=A_{0}+A_{1}\exp\left(-t/t_{1}\right)+A_{2}\exp\left(-t/t_{2}\right)+A_{3}\exp\left(-t/t_{3}\right)$$

(2) $$\tau_{\mathrm{avg}}=\frac{A_{1}t_{1}+A_{2}t_{2}+A_{3}t_{3}}{A_{1}+A_{2}+A_{3}}$$

where *A_i_* (*i* = 0,1,2,3) is the amplitude of the PL decay for *i*-component, *t* is the decay time component. Fit residuals are presented in Figure S2A–C. The dependence is shown in Figure 1D. The PL lifetimes shorten with temperature decreasing as previously ascribed to the effect of giant oscillator strength transition [29,38]. This hypothesis is supported by the growth of PL intensity (inset in Figure 1D) with lowering temperature. The gradual reduction of PL lifetimes with temperature ends with some saturation at low temperatures, which can be explained by the competing contribution of long-lived trap-related radiation, whose input into the total PL signal increases with decreasing temperature. Vice versa, the general trend of a decrease in the PL intensity with increasing temperature is accompanied by an increase in intensity for the highest temperatures, which can be explained by the thermal release of trapped carriers with subsequent radiative recombination.

**Figure 2:**
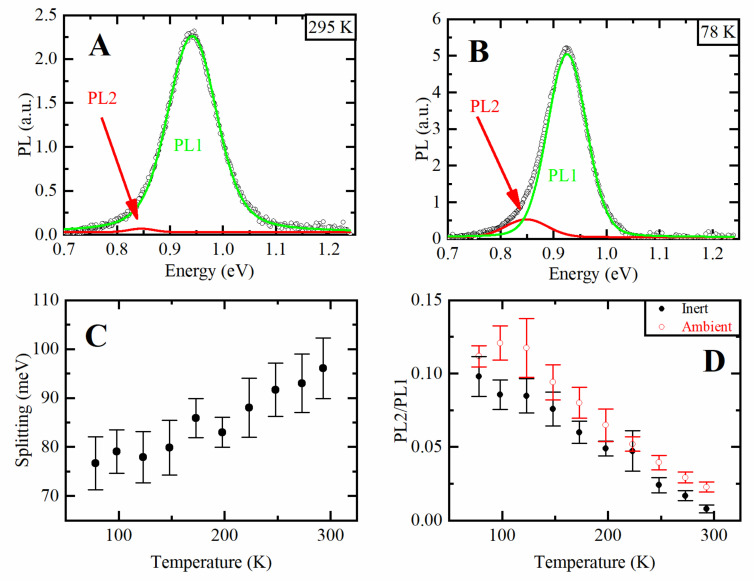
PL spectra of 4 ML PbSe NPLs at (**A**) 295 K and (**B**) 78 K, green line stays for PL1, red line—for PL2; (**C**) energy splitting between PL1 and PL2 peak positions; (**D**) ratio of PL2 to PL1 areas, black dots—NPLs purified in inert atmosphere, red dots—NPLs purified in ambient atmosphere.

PL spectra recorded at different temperatures are shown in the inset in Figure 1C. PL spectrum at 78 K demonstrates noticeable asymmetry. The PL spectra can be described by a sum of two peak functions as shown in Figure 2A,B, we then label the high-energy peak as PL1 and attribute it to the band-edge emission. This peak is described by a Voight function [49]. The low-energy peak is labeled as PL2 and can be fitted by a Gaussian function. Fit residuals are shown in Figure S2D,E. Interestingly, both the energy splitting between two peaks and the ratio of their amplitudes depend on temperature, as shown in Figure 2C,D. Asymmetry was earlier observed for PbSe QDs and ascribed to intervalley splitting, and the splitting energy was dependent on QD size and temperature, namely the splitting energy increased when temperature decreased [50]. Temperature-dependent amplitude ratio between band-edge and trap-related PL was observed for CdTe QDs in NaCl matrix: the contribution from low-energy trap-related emission increased with temperature decrease [51]. In our case, low-energy emission became more pronounced at low temperatures, and the splitting energy decreases with temperature. That may indicate that the PL2 can be ascribed to trap-related emission. Importantly, we compared two samples of PbSe NPLs processed for further measurements (precipitation, washing) in an inert and ambient atmosphere. For both samples we observed the same temperature dependencies of PL2/PL1 ratio. However, at any temperature the relative contribution from PL2 was larger for the NPLs precipitated in ambient environment, that may indicate the influence of oxygen and moisture which increases the density of luminescent trap states.

An appearance of more pronounced additional peak in PL spectra has been recently observed in CdSe NPLs at low temperatures in several studies. Here we do not consider such effects as a phonon replica [31] or bright-dark exciton splitting [35], which are usually observed at cryogenic temperatures. Erdem et al. have recently found an additional emission at temperatures below 180 K [33]. It is important to note that the authors reported the increase of the low-energy emission contribution when the temperature decreased. van der Bok et al. studied the PL in the temperature range of 4–423 K and reported an extra peak appeared on the low energy side upon cooling [36]. Yu et al. studied the NPLs of different lateral sizes and observed the surface trap-related PL at *T* < 200 K, which can be even more intensive than the band-edge related one [52]. The energy difference between the two peaks was not dependent on temperature, but the contribution from low-energy emission increased for NPLs with smaller lateral dimensions. To confirm the surface trap-related origin of this emission, the authors passivated the surface with polydimethylsiloxane and demonstrated suppressed low-energy emission at low temperature.

Thus, the experimental and literature data allow us to conclude that the PL2 emission is associated with surface-related trap states. In our case, a large FWHM may mask a distinct splitting onto two peaks. This is typical for QDs where a size distribution contributes into inhomogeneous broadening and the splitting looks like asymmetry and broadening of PL [27,53]. van der Bok et al. reported asymmetry of PL from CdSe/CdS NPLs instead of splitting and ascribed it to the larger inhomogeneous broadening induced by a shell growth [36].

**Figure 3:**
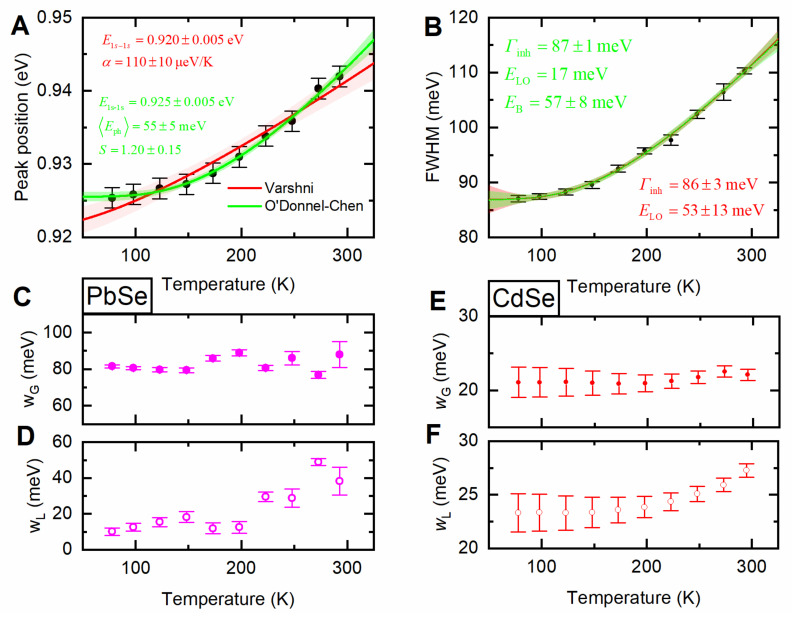
(**A**) PL1 peak position of four ML PbSe NPLs vs. temperature, the red line marks the Varshni fit, the green line marks the O’Donnel-Chen fit, and the semi-transparent areas mark the 95% confidence band; (**B**) PL1 full width at half maximum (FWHM) vs. temperature, lines show the FWHM fit with Equation (3) (red) and Equation (4) (green), and the semi-transparent areas mark the 95% confidence band; (**C**,**D**) PbSe (purple) and (**E**,**F**) CdSe (red) Gaussian (solid), and Lorentz (open) linewidths extracted from the Voight fitting.

Now we should consider PL1 which was fitted by a Voight function in more detail. The temperature dependence of the bandgap was previously studied for PbSe QDs using both absorption [53] and PL [54] spectroscopies. It was shown that the coefficient of temperature shift is size-dependent and changes from negative (for the smallest QDs) to positive for larger QDs. Assuming the PL1 is related to the band-edge emission, the PbSe NPLs under study demonstrate an increase of bandgap with temperature as shown in Figure 3A. The coefficient of a temperature PL shift was obtained by Varshni fitting [55] and equals to 110 ± 8 µeV/K, which is close to that obtained for 4.6 nm PbSe QDs. O’Donnel function [56] allows for more precise approximation of the obtained experimental data, giving the average phonon energy of 55 ± 5 meV.

FWHM of the PL1 is shown in Figure 3B. The temperature dependence of the FWHM is often described by the following equation:

(3) $$\mathrm{FWHM}(T)=\Gamma_{\mathrm{inh}}+\sigma T+\gamma / [\exp\left(E_{\mathrm{LO}}/k_{B}T\right)-1]$$

where *Γ*_inh_ is the temperature independent inhomogeneous broadening constant, *σ* is the exciton-acoustic phonon coupling coefficient, *γ* is the temperature-independent linewidth parameter characterizing the total linewidth due to exciton-LO-phonon (longitudinal-optical) interactions, and *E*_LO_ is the LO-phonon energy [26,57]. The fitting gives the values of *Γ*_inh_ = 86 ± 3 meV and *E*_LO_ = 53 ± 13 meV. The coupling coefficient for acoustic phonons is several orders smaller than that obtained for the interaction with optical phonons which is typical for the studied temperature range [58] and indicates the strong Fröhlich exciton-photon interaction. Although a large LO phonon energy of 37–43 meV was previously reported for PbSe QDs [59], the fitted value is too far from that for the bulk PbSe (17 meV). Indeed, the Raman spectroscopy measurements (see Figure S3 and Table S1, Supplementary Materials) performed for the PbSe NPLs give the LO phonon frequency of 150 cm^−1^ (18.6 meV). For more careful approximation of FWHM temperature dependence, several effects, such as the interaction of excitation with impurities [57] and bound excitons [60], can be considered. The contribution from bound excitons would not be pronounced in the studied temperature range. The interaction with impurities can be presented by introducing an additional term to Equation (3). To reduce the number of varied parameters during fitting, we neglected the weak interaction with acoustic phonons and kept the *E*_LO_ equal to experimentally determined 18.6 meV, then the expression might be written as follows:

(4) $$\mathrm{FWHM}(T)={Ɖ}_{\mathrm{inh}}+\varphi_{\mathrm{imp}}\exp [-E_{B}/k_{B}T]+\gamma / [\exp\left(E_{\mathrm{LO}}/k_{B}T\right)-1]$$

where *φ*_imp_ is the impurity broadening coupling constant, and *E_B_* is the averaged binding energy of the impurities. The fitting gives *Γ*_inh_ = 87 ± 1 meV and *E*_B_ = 57 ± 9 meV.

More careful consideration of the FWHM temperature dependence includes the study of the dependencies for the contribution of the Gaussian and Lorentz components included in the convolution of the Voigt function. The Gauss component is usually ascribed to inhomogeneous broadening, while the Lorentz one describes the homogeneous broadening. Figure 3C,D shows the temperature dependencies obtained for the two components. As expected, the Gaussian component does not depend on temperature, and the medium value is 82 ± 4 meV which is close to the *Γ*_inh_ = 87 ± 1 meV value obtained from the fitting of a total FWHM for the PL1. The Lorentz component narrows with temperature decrease from ~40 to ~10 meV. This behavior is similar to that demonstrated by the parent CdSe NPLs (Figure 3E,F and Figure S4 (Supplementary Materials)). van der Bok et al. have recently shown a linear temperature dependence of homogeneous broadening for CdSe NPLs with a constant contribution from the inhomogeneous one [36]. Thus, PbSe and CdSe NPLs demonstrate similar values and dependencies of homogeneous PL broadening but very different FWHM. In other words, a widening of the PbSe PL spectrum is not associated with the internal mechanisms of emission but is associated with an increase in width due to inhomogeneous broadening.

## 4. Discussion

Several reasons should be considered that can lead to an increase in the inhomogeneous broadening of the NPLs’ PL spectrum. The first contribution to the PL broadening comes from the morphological considerations which include the size, shape, and geometry of the nanoparticles. Broad PL spectra of our PbSe NPLs may be attributed to a wide distribution of their lateral sizes, as clearly seen from their TEM image in Figure 1B. Small irregularly-shaped species present in the sample may behave as a 0D confined system, i.e., QDs, rather than 2D, due to a large Bohr radius of PbSe (46 nm) [61]. Khan et al. have recently shown that a change of the lateral size of PbS NPLs allows for a slight tuning of their PL peak position [11]. However, it should be also pointed out that wide PL bands were observed for 50–100 nm PbSe NPLs [16] and even µm-sized PbS sheets [9,10]. Additionally, the specimens can contain a certain number of smaller nanoparticles with properties closer to QDs rather to NPLs. For these byproducts, the quantum confinement effect may have a more pronounced impact and lead to some size-distribution of optical responses. The influence of size [50,62,63] and size distribution [64,65,66] of lead chalcogenides QDs on their PL properties have been well established in recent years. On the other hand, such fractions should have their own statistical size-distribution, which will lead to different additional contributions to NPLs’ PL at different wavelengths. In this regard, uniform broadening of the main product PL spectrum seems unlikely. Further, individual NPLs can form local ensembles due to the strong van der Waals interaction. In this case, their optical properties will be disturbed by the process of nonradiative energy transfer within the ensemble [67,68,69], which should be especially pronounced for such a geometry of the nanostructure [70]. Additional aggregation can occur during their infiltration into a porous matrix or poly(methyl methacrylate). Despite a low NPLs concentration and thorough homogenization, some fraction of aggregated NPLs can form during the samples preparation. In any case, to clarify all these morphological contributions, further investigation of the PL properties of Pb chalcogenide-based NPLs, including the single particle spectroscopy, is required.

Second, the quality of the crystal lattice must be taken into account. For instance, the structural disorder was reported to play a crucial role in line broadening observed in semiconductor quantum wells [71]. Recently, Janke and co-authors performed a detailed study on InP QDs and demonstrated that internal lattice defects induced by chemical modification are responsible for a drastic increase of PL inhomogeneous broadening [72]. The structural disorder or internal lattice defects in PbSe NPLs can arise from rearrangements of the atoms during the cation exchange reaction. An interesting parallel can be drawn with PbSe QDs, for which a larger FWHM of the PL band is usually observed for the QDs obtained by the cation exchange method, rather than by the direct hot-injection synthesis (see Table S2, Supplementary Materials). It is also worth noting that wide PL bands are observed for the Pb chalcogenide-based NIR-emitting NPLs [16], nanosheets [9,10], and nanoribbons [12] obtained by the oriented attachment method. To verify this hypothesis, we performed the additional study of FWHM dependencies of PL and Raman scattering bands for PbSe QDs of the same size achieved by a direct hot-injection synthesis and cation exchange method. SEM-image of the cation-exchanged PbSe QDs are shown in Figure S1D (Supplementary Materials). The temperature dependencies of PL parameters observed in PbSe QDs obtained by the cation exchange method are qualitatively identical to those obtained for PbSe NPLs (see Figure S5, Supplementary Materials). Interestingly, the observed temperature-dependent homogeneous broadening presented by the Lorentzian part of the approximation demonstrates similar to the PbSe NPLs behavior, both qualitatively and quantitatively. The PbSe QDs prepared by hot-injection and cation exchange procedures exhibit very similar optical responses, including the coefficient of PL peak position temperature shift (Figure S5, Supplementary Materials). However, the QDs obtained by cation exchange show noticeably wide PL band. The temperature-independent mean value of the Gaussian part, which is responsible for inhomogeneous broadening, was found to be ~110 meV for cation exchanged PbSe QDs, which is ~20 meV larger than that determined for the QDs synthesized by the direct hot-injection method. However, Raman spectra obtained for the PbSe QDs of the same size, synthesized by cation-exchange and direct hot-injection methods, demonstrate similar behavior. The positions of phonon peaks were determined and compared to the known literature data [73,74,75,76]. The positions of the typical phonon peaks (LA(X), 2TA(X), LO(X) and/or LA(X)/TA(X), 2TO(X), and LO(Γ)) are close for both types of QDs and PbSe NPLs (see Table S1, Supplementary Materials). Although the bandwidth associated with 2TO(X) and LO(X) and/or LA(X)/TA(X) phonons is larger for the QDs obtained by the cation exchange method, the bandwidth associated with the LO(Γ) phonons was larger for the QDs obtained by the direct hot-injection method. Thus, the Raman spectra do not demonstrate significant broadening caused by the distortion of the crystal lattice during cation exchange. 

The third contribution to PL line broadening of the PbSe NPLs may come from the presence of surface defect induced trap states. It was shown that the PL properties (including FWHM) are sensitive to surface modification, including shell growth [77,78,79,80] and ligand manipulation [80]. The surface-defect related shallow traps can form a continuum of sublevels that will contribute to the emission and lead to its broadening. The influence of ligands on optical and electrical properties, stability, and self-organization processes have been well studied for 0D Pb chalcogenide-based systems [81,82,83,84], that, unfortunately, cannot be said about their 2D counterparts. To assess this contribution, more research is needed on the surface chemistry of PbSe and PbS NPLs. Together with the temperature study of single NPLs PL, it will provide a deeper understanding of the mechanisms underlying the inhomogeneous broadening of the PL from Pb chalcogenide-based NPLs.

## 5. Conclusions

To conclude, we performed a comprehensive PL study of four ML PbSe NPLs prepared by the cation exchange method. We have demonstrated that besides the band-edge related emission from the lowest excitonic state, NPLs possess additional red-shifted emission band, whose spectral position and intensity depend on temperature. We showed that this emission is more pronounced for air-processed NPLs, which proves its surface-trap related nature. We have revealed that wide FWHM of the band-edge NPLs’ PL is attributed to the inhomogeneous broadening, while the homogeneous broadening of PbSe NPLs is close to those observed for CdSe NPLs and PbSe QDs. The possible mechanisms of inhomogeneous PL broadening are described.

## Supplementary Materials

The following are available online at https://www.mdpi.com/2079-4991/10/12/2570/s1, Materials and methods, Figure S1: TEM images of NPLs and QDs, Figure S2: Fit residuals for NPLs PL decay and PL spectra, Figure S3: Raman spectra for PbSe QDs and NPLs, Figure S4: CdSe NPLs PL data, Figure S5: PbSe QDs PL data, Table S1: Assessment of transitions in Raman spectra, Table S2: PL parameters for PbSe QDs.
